# Exposure to artificial light at night alters innate immune response in wild great tit nestlings

**DOI:** 10.1242/jeb.239350

**Published:** 2021-05-14

**Authors:** Ann-Kathrin Ziegler, Hannah Watson, Arne Hegemann, Richard Meitern, Virginie Canoine, Jan-Åke Nilsson, Caroline Isaksson

**Affiliations:** 1 Department of Biology, Lund University, 223 62 Lund, Sweden; 2 Department of Zoology, University of Tartu, 51005 Tartu, Estonia; 3 Department of Behavioural and Cognitive Biology, University of Vienna, 1090 Vienna, Austria

**Keywords:** ALAN, Immune function, Light pollution, Melatonin, Physiology, Urbanization

## Abstract

The large-scale impact of urbanization on wildlife is rather well documented; however, the mechanisms underlying the effects of urban environments on animal physiology and behaviour are still poorly understood. Here, we focused on one major urban pollutant – artificial light at night (ALAN) – and its effects on the capacity to mount an innate immune response in wild great tit (*Parus major*) nestlings. Exposure to ALAN alters circadian rhythms of physiological processes, by disrupting the nocturnal production of the hormone melatonin. Nestlings were exposed to a light source emitting 3 lx for seven consecutive nights. Subsequently, nestlings were immune challenged with a lipopolysaccharide injection, and we measured haptoglobin and nitric oxide levels pre- and post-injection. Both haptoglobin and nitric oxide are important markers for innate immune function. We found that ALAN exposure altered the innate immune response, with nestlings exposed to ALAN having lower haptoglobin and higher nitric oxide levels after the immune challenge compared with dark-night nestlings. Unexpectedly, nitric oxide levels were overall lower after the immune challenge than before. These effects were probably mediated by melatonin, as ALAN-treated birds had on average 49% lower melatonin levels than the dark-night birds. ALAN exposure did not have any clear effects on nestling growth. This study provides a potential physiological mechanism underlying the documented differences in immune function between urban and rural birds observed in other studies. Moreover, it gives evidence that ALAN exposure affects nestling physiology, potentially causing long-term effects on physiology and behaviour, which ultimately can affect their fitness.

## Abstract (precis)

**Summary:** Exposure to artificial light at night changes aspects of baseline innate immunity and the response towards an immune challenge in wild great tit nestlings, which is probably mediated by reduced levels of melatonin.

## INTRODUCTION

The natural light regime functions as one of the most reliable and consistent environmental cues, orchestrating many evolutionary and ecological processes. Annual changes in daylength have been shown to entrain stages in the annual cycle such as migration and reproduction across taxa (Gwinner, 1986; Nelson et al., 2010). This natural light is a Zeitgeber, which additionally allows organisms to synchronize their physiological processes such as metabolism and immune function (Majewski et al., 2012; Markowska et al., 2017) on a diel time scale (Dunlap, 1999), leading to adaptive regulation of body functions. The extensive use and installation of electrical night lighting during the last century has become an increasing concern and been recognized as a stressor for organisms living in anthropogenic environments, because of its potentially disruptive effects on physiology and behaviour (Dominoni et al., 2013a; Gaston et al., 2013; Navara and Nelson, 2007; Ouyang et al., 2018; Swaddle et al., 2015). Shifts in the timing of certain behaviours, e.g. dawn singing and foraging (Kempenaers et al., 2010; Santos et al., 2010) and migration (Van Doren et al., 2017), could expose individuals to a higher risk of predation and lead to a mismatch with environmental conditions such as food abundance during offspring rearing.

The hormone melatonin has been shown to act as a crucial mediator of natural dark–light cycles, as its release (mainly from the pineal gland) follows and synchronizes daily rhythms in response to light conditions (Gwinner et al., 1997). This responsiveness to daylight leads to high concentrations of melatonin in blood during darkness, which, for example, regulates sleep rhythms (reviewed in e.g. Reiter et al., 2010). Exposure to even low intensities of artificial light at night (ALAN), as in urban environments, has been shown to disrupt circadian rhythms over a broad range of taxa by suppressing or reducing the release of melatonin (de Jong et al., 2016; Dominoni et al., 2013a; Grubisic et al., 2019). Specifically, this change in melatonin concentration, and hence disruption of circadian rhythm, has been linked to a multitude of behavioural changes and health effects (Navara and Nelson, 2007; Swaddle et al., 2015) ranging from changes in the timing of singing and reproduction in different songbirds (Dominoni et al., 2013b; Kempenaers et al., 2010; Miller, 2006) to cancer growth (Stevens et al., 2007), obesity, depression and metabolic syndrome in rodents and humans (reviewed in e.g. Fonken and Nelson, 2014; Logan and McClung, 2019).

Variation in physiology in the presence of ALAN is attributed to the diverse functional role of melatonin, possessing not only a circadian pacemaker function but also antioxidant and immunomodulatory properties (reviewed in e.g. Carrillo-Vico et al., 2005; Reiter et al., 2010). It is well established that melatonin is instrumental in the bidirectional relationship between the pineal gland and the immune system (e.g. Calvo et al., 2013; Markus and Ferreira, 2011). Pinealectomy, and thereby reduced melatonin levels, results in delayed development of immune tissues and function in birds (Jankovíc et al., 1994). Exposure to ALAN in laboratory settings suppresses immune responses in rodents (Bedrosian et al., 2011; Oishi et al., 2006), birds (Moore and Siopes, 2000) and invertebrates (Durrant et al., 2020). Moreover, administration of exogenous melatonin reverses or mitigates the detrimental effects of ALAN on the immune system (Jones et al., 2015; Moore and Siopes, 2000). Accumulating results indicate a tight circadian control of the immune system, including regulating the activity and controlling the movement of innate immune cells (reviewed in Scheiermann et al., 2018). Specifically, experimental ALAN exposure led to the abolishment of rhythmic expression of immune genes in different tissues of zebra finches, *Taeniopygia guttata* (Mishra et al., 2019). Thus, the disruption of the diel physiological homeostasis by ALAN is likely to negatively affect health and could ultimately decrease fitness. Indeed, exposure to ALAN supressed melatonin levels and increased mortality in house sparrows, *Passer domesticus*, that were infected with West Nile virus (Kernbach et al., 2020).

While the effects of light on the interaction between endocrine and immune mechanisms have been examined extensively under laboratory conditions, studies in wild animals are still sparse. Baseline innate immune markers have been compared between wild populations in rural versus urban habitats, although in these studies light pollution was only one of many urban stressors (e.g. Bailly et al., 2017; Capilla-Lasheras et al., 2017; Watson et al., 2017). Raap et al. (2016a) found altered baseline innate immune markers after an experimental ALAN exposure of wild great tit, *Parus major*, nestlings during two nights. While the effects of the urban environment and more specifically light pollution on baseline immune parameters have been considered, effects on the innate immune response have been far less studied. One study found that the urban habitat negatively affected the immunity of great tit nestlings (Bailly et al., 2016), but another found no effects on the immune response of amphibians (Iglesias-Carrasco et al., 2017). While maintaining baseline innate (constitutive) immune function is a vital part of the defence against pathogens invading the body, the capacity to mount an adequate innate immune response is also crucial for survival once a pathogen starts replicating in the body. Such an immune response comes with substantial behavioural and physiological costs (Bonneaud et al., 2003; Burness et al., 2010; Hart, 1988; Hegemann et al., 2018). Hence, though closely related, baseline constitutive immune function and induced immune responses need to be carefully separated (Hegemann et al., 2013a; Vermeulen et al., 2016).

To the best of our knowledge, no study has yet experimentally tested the effects of ALAN exposure on both baseline innate immune function and the ability to mount an innate immune response in wild birds. The innate immune system plays a crucial role, especially during postnatal development when the acquired branch of the immune system is not yet fully developed in young animals (Grindstaff et al., 2006; Lawrence et al., 1981). The early-life period of an animal might be particularly sensitive to adverse environmental influences, which could change developmental trajectories and have implications for adult physiology and behaviour (Gluckman et al., 2005). Early-life lighting conditions have been found to affect the development of the circadian system (Brooks and Canal, 2013; Fonken and Nelson, 2016), with effects on, for example, food intake (Cissé et al., 2017), fear response (Borniger et al., 2014), body mass (Raap et al., 2016b), growth rate and survival (O'Connor et al., 2019).

In the present study, we experimentally investigated the effects of ALAN exposure on both the baseline innate immunity and the capacity to mount an innate immune response. We mimicked a bacterial infection by triggering an immune response with a lipopolysaccharide (LPS) injection in wild great tit nestlings and assessed the magnitude of the innate immune response by measuring circulating haptoglobin and nitric oxide levels before and after the LPS injection of both ALAN- and dark-treated nestlings. LPS is an endotoxin found in the cell walls of most gram-negative bacteria, which elicits an acute-phase immune response resulting in an inflammatory reaction (Owen-Ashley and Wingfield, 2007). The acute-phase response is mediated by cytokines and chemokines, which are generated by immune-competent cells such as macrophages (Cray et al., 2009; Klasing, 1998). Additionally, an oxidative burst is initiated, leading to high levels of pro-oxidant molecules, and acute-phase proteins are synthesized in the liver in order to fight the invading pathogen. Haptoglobin is an acute-phase protein, widely used as a marker for the intensity of the inflammatory response (Matson et al., 2012; Quaye, 2008). Under constitutive conditions, it is found at low concentrations in the blood, but in response to an acute pathogenic threat, concentrations increase rapidly (Millet et al., 2007; van de Crommenacker et al., 2010). Functionally, haptoglobin prevents oxidative damage by binding free haemoglobin released from lysed red blood cells. Nitric oxide is a multifunctional signalling molecule involved in the modulation of inflammatory processes and direct destruction of pathogens (Bogdan, 2001; Coleman, 2001; Sild and Hõrak, 2009). It is produced by activated macrophages and other immune-system cells or induced by endotoxins and reactive oxygen species (Wink et al., 2011). Similar to haptoglobin, nitric oxide circulates in low concentrations during baseline conditions, but can increase severalfold after an immune challenge (Bourgeon et al., 2007; Sild and Hõrak, 2009). We predicted that ALAN exposure, via its disruptive effects on physiological rhythms and expected reduction of melatonin levels, would lead to a lower baseline concentration of haptoglobin and nitric oxide than in dark-night nestlings. Moreover, we expected that ALAN exposure would weaken the innate immune response to the immune challenge, resulting in lower circulating haptoglobin and nitric oxide concentrations in ALAN-exposed birds than in dark-night birds following immune challenge. Additionally, we quantified body mass loss as a consequence of the immune challenge and expected ALAN-exposed birds to lose less body mass overnight as a result of a weaker sickness response than dark-night nestlings. Lastly, we predicted that ALAN-exposed nestlings would have a reduced growth rate compared with dark-night nestlings over the course of the experiment, because of the role of melatonin in cytoskeletal modulation (Benítez-King, 2006).

## MATERIALS AND METHODS

The study was performed during the breeding season of 2018 (20 May to 08 June) in Southern Sweden in the forest of Skrylle Nature Reserve (55°41′33″N, 13°21′36″E) using a nest box population of free-living great tits. The forest is composed of mixed and deciduous forest. Sunrise and sunset at the start of the experiment were at 04:47 h and 21:20 h and, at the end, at 04:25 h and 21:47 h, respectively. This study was approved by the Malmö/Lund ethical committee (permit no. 04706/2018).

### Experimental set-up and sampling

Nest boxes were checked once a week from early April to determine lay date, clutch size and onset of incubation. From day 11 of incubation, nests were checked daily to determine exact hatching date. Thirty-eight occupied nest boxes were assigned to one of the two treatment groups: ALAN exposure (*N*=19) or dark-night (no ALAN exposure, *N*=19). Between two and four new nests were added to the experiment each day, alternating between dark-night and ALAN exposure treatment. The ALAN nest boxes were exposed to a light source for seven consecutive days (days 7–14 post-hatching), which consisted of a small LED light (5 mm, warm white, 2700–3000 K, art. no. 90734, Kjell & Company) placed under the nest box roof. The diodes were standardized to produce 3 lx at a distance of about 20 cm in the nest box (measured with a light meter, LM-120, Amprobe), which approximately corresponds to the position of the nestlings. We chose this light intensity as ecologically relevant, because similar levels have been shown in urban environments (Dominoni, 2017). The LEDs were installed at day 7 post-hatching (between 11:45 h and 19:30 h) and were left on permanently until they were removed at day 14. Because LEDs were used, there was no warming effect inside the nest boxes. The two experimental groups were treated the same: dark-night boxes also received LED lamps, but the lights in the dark-night nest boxes were never switched on. The mean brood size on day 7 for the dark-night and ALAN treatment group was 7.16±0.34 and 7.68±0.29 nestlings (mean±s.e.m.), respectively (total dark-night *N*=136, ALAN *N*=146), and did not differ between the experimental categories at the start of the light manipulation (*t*-test: *t*=−1.192, *P*=0.2). At day 7, all nestlings were ringed with an aluminium ring, and body mass was measured with a Pesola spring balance (±0.1 g). Body mass measurements were repeated on day 9, 11, 13 and 14 with an additional measurement of wing length on day 13 and 14.

At day 13, a blood sample was taken from all nestlings (between 23:27 h and 02:20 h, dark hours) from the jugular vein. Each brood was randomly split into two groups, by alternately assigning every nestling to one of the two sampling groups. From one group of nestlings, we collected ∼100 µl of blood for later analysis of melatonin levels (see below), whereas from the second group we collected ∼60 µl blood for later analysis of immune response markers (see below). After blood sampling, this second group of the brood was additionally subjected to an immune challenge, where they received a subcutaneous injection of 17 µg LPS suspended in 40 µl of phosphate-saline buffer (individual dosage: 1 µg LPS g^−1^ body mass, LPS from *Escherichia coli* 055:B5, Sigma-Aldrich) to induce an acute-phase immune response (Bonneaud et al., 2003; Owen-Ashley and Wingfield, 2007). During the sampling, we used head torches with white light and sampling took on average 19.6 min per nest, with on average 2.75 min per nestling. At day 14 (between 14:21 h and 18:09 h, on average 15 h 29 min ±19 min after the LPS injection), a second blood sample (∼60 µl) was taken from those nestlings that had received the LPS injection to quantify the within-individual change in immune responses. All blood samples were kept on ice for a maximum of 4 h, then centrifuged at 6000 rpm for 10 min to separate the plasma and stored at −80°C for later analysis. We alternated the order of sampling between treatments every evening to avoid a bias in sampling time between the ALAN and dark-night broods.

### Immune assays

A commercially available colorimetric kit (TP801, Tri-Delta Diagnostics, Morris Plains, NJ, USA) was used to quantify plasma haptoglobin concentration (mg ml^−1^). This functional assay quantifies the haem-binding capacity of plasma. We followed the ‘manual method’ provided by the manufacturer with a few minor modifications (following Hegemann et al., 2012; Matson et al., 2012). Absorbance was measured at two wavelengths (405 and 450 nm) prior to the addition of the final reagent that initiated the colour-change reaction to be able to correct for differences in plasma redness, an indicator of haemolysis, which can affect the assay (Matson et al., 2012).

Nitric oxide concentration was assessed by spectrophotometrically quantifying the concentration (µmol l^−1^) of stable oxidation end-products of nitrate (NO_3_^−^) and nitrite (NO_2_^−^) in plasma. We followed the protocol described in Sild and Hõrak (2009), with the modification of 15 µl plasma and the respective adjustment of buffer volume. Measurement repeatability (Lessells and Boag, 1987) for eight blood samples run in duplicate or triplicate, depending on the amount of plasma, was 0.79 (*F*_7,9_=8.75, *P*=0.002).

### Melatonin analysis

The plasma concentration of melatonin (pg ml^−1^) was measured by direct radioimmunoassay (RIA) following chloroform extraction (details in Fusani and Gwinner, 2004; Goymann et al., 2008). Titriated melatonin was purchased from Perkin Elmer (NET801) and melatonin antiserum from Stockgrand (G/S/704-8483). Samples were analysed in two assays. The intra-assay coefficient of variation calculated from a series of controls was 4.2% and 3.3%, respectively, for the two assays, whereas the inter-assay coefficient of variation based on the same controls was 11%. Melatonin concentration was adjusted for average recovery (85%). The detection limit of the assay was 15.6 pg ml^−1^.

### Statistical analyses

We only included nestlings that reached day 14 in the statistical analysis and additionally excluded one dark-night nest from the analysis, because all but two of the nine nestlings died during the experimental period (dark-night *N*=124, ALAN *N*=143). Because of plasma limitations, sample size varied between assays and time points.

All analyses were run in R 3.6.3 (http://www.R-project.org/). We performed stepwise backwards elimination of factors with *P*>0.1 for the linear mixed models (LMM, R package ‘lmerTest’; Kuznetsova et al., 2017), starting with the least significant highest order term. Random effects and ALAN exposure treatment (i.e. dark-night versus ALAN exposure) were always retained in the model. Normality of residuals was visually checked. All numerical covariates were centred to facilitate interpretation of the estimates. For all models, we used the Satterthwaite approximation to calculate the denominator degrees of freedom and *P*-values. *Post hoc* pairwise comparisons were performed using estimated marginal means (R package ‘emmeans’; https://github.com/rvlenth/emmeans). We present means±s.e.m., if not stated otherwise.

To test the effect of ALAN exposure on plasma melatonin concentration, we used a LMM including the ALAN exposure treatment as a fixed effect (dark-night versus ALAN), body mass at day 13 and hatching date as covariates, together with the interaction between body mass at day 13 and ALAN exposure treatment. Furthermore, nest ID was included as a random effect. Melatonin concentration was log-transformed to achieve normality of residuals.

The effects of ALAN exposure on the capacity to mount an immune response were tested with LMMs by comparing haptoglobin and nitric oxide before and after LPS injection. For both models, we included, as fixed effects, time point of sampling (i.e. pre- or post-immune challenge), ALAN exposure treatment (dark-night versus ALAN) and the interaction between the two factors. Body mass measured on day 13 (i.e. pre-immune challenge) and hatching date were used as covariates. Additionally, absorption of the plasma measured at 450 nm, i.e. intensity of plasma redness (Matson et al., 2012), and the interaction between plasma redness and the time point of sampling were included as covariates in the haptoglobin model. This interaction was included because of a greater variation in plasma redness in the post-immune challenge samples, which is probably caused by higher rates of haemolysis as a result of the immune challenge (Brauckmann et al., 2016). As random effects, we included nestling ID nested within nest ID to account for non-independence of siblings and repeated measurements. However, for the haptoglobin model, this random effect structure resulted in singular fit issues, so we decided to include a simpler random effect structure with only nest ID. A comparison with a likewise built Bayesian Markov chain Monte Carlo generalized linear mixed model (MCMCglmm, R package ‘MCMCglmm’; Hadfield, 2010) revealed similar results. We used a Pearson's correlation test to analyse the association between haptoglobin and nitric oxide concentrations for pre- and post-immune challenge, respectively.

The effect of ALAN exposure on nestling mass over time, i.e. growth rate, was estimated with a LMM, where body mass on day 7, 9, 11 and 13 was used as the fixed effect. We used ALAN treatment, age (centred, i.e. numerical day 7, 9, 11 and 13), age^2^ and hatching date as covariates and also included the two interactions between ALAN exposure treatment and the two age variables. As random effects, we included nestling ID nested within nest ID.

In order to assess the effects of ALAN exposure on nestling biometrics (all nestlings included: body mass and wing length on day 13), we fitted LMMs with ALAN exposure treatment as a fixed effect and hatching date as a covariate. Nest ID was included as a random effect. The effects of ALAN exposure and the subsequent LPS injection on nestling biometrics (only immune-challenged nestlings included: body mass and wing length pre- and post-immune challenge) were analysed by fitting LMMs using ALAN exposure treatment, time point of measurement (i.e. pre- and post-immune challenge) and the interaction between the two variables as fixed effects. Hatching date was used as a covariate and nestling ID nested within nest ID was included as a random effect to account for non-independence of siblings and repeated measurements.

## RESULTS

### Physiological markers

**Fig. 1.**
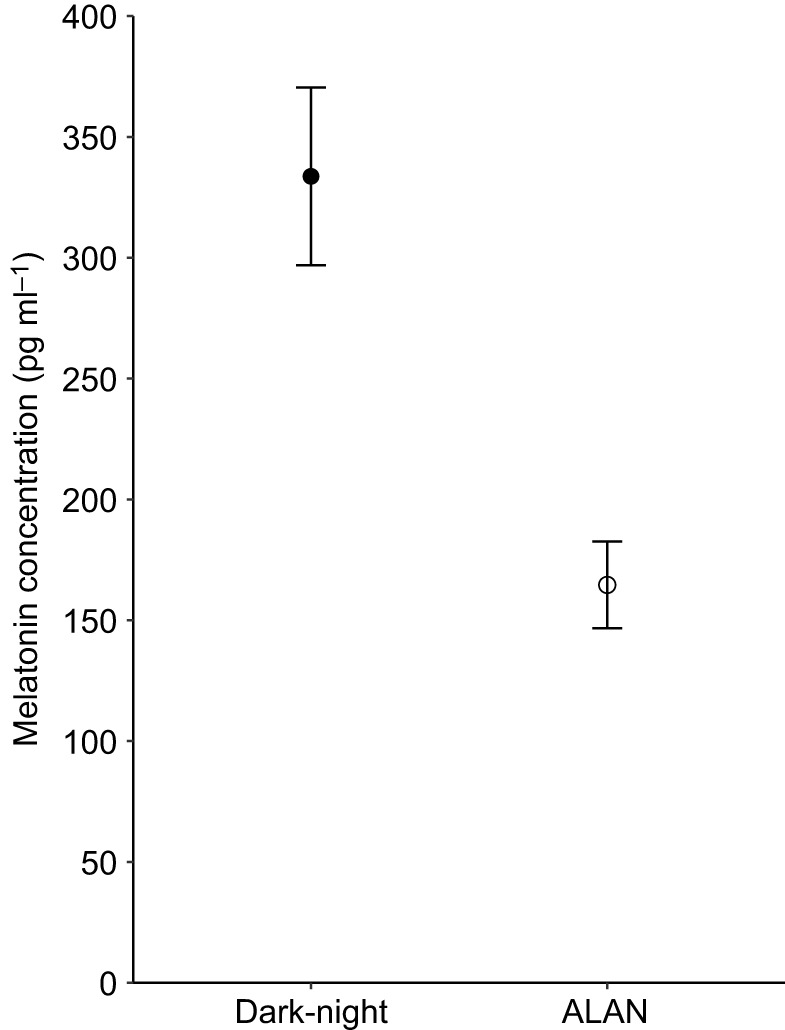
**Effect of exposure to artificial light at night (ALAN) on melatonin concentration in plasma of great tit nestlings.** Nestlings were assigned to dark-night (*N*=50) or ALAN (*N*=50) treatment. Back-transformed estimated marginal means±s.e.m. calculated from the final model (Table 1) are shown.

**Fig. 2.**
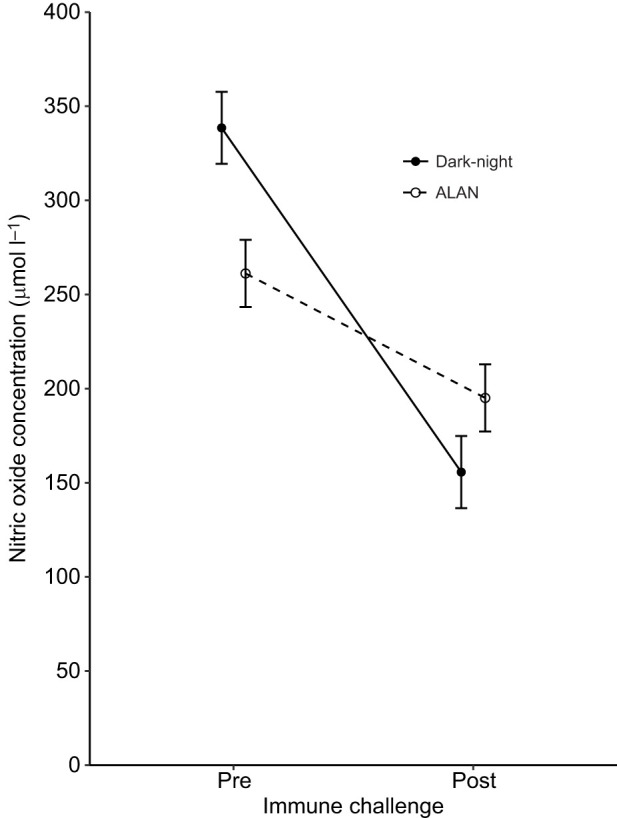
**Effect of exposure to** **ALAN and immune challenge on nitric oxide concentration in plasma of great tit nestlings.** Nestlings were assigned to dark-night (*N*=61) or ALAN (*N*=76) treatment and then subjected to an immune challenge (LPS injection). Estimated marginal means±s.e.m. calculated from the final model (Table 1) are shown.

ALAN-exposed nestlings showed on average 49% lower plasma melatonin concentrations than dark-night birds (199.3±18.9 versus 404.7±33.7 pg ml^−1^, respectively; ALAN *N*=50 nestlings of 19 broods, dark-night *N*=50 nestlings of 18 broods; Table 1, model 1; Fig. 1). We also found a significant interaction between the immune challenge and ALAN exposure treatments on plasma nitric oxide concentration (ALAN *N*=76 nestlings of 19 broods, dark-night *N*=61 nestlings of 18 broods; Table 1, model 2; Fig. 2). Dark-night birds showed a steeper decline in the concentration of nitric oxide as a response to the immune challenge compared with ALAN-exposed nestlings (Table 1, model 2; Fig. 2). In line with our prediction, *post hoc* pairwise comparisons showed that ALAN exposure significantly decreased baseline nitric oxide levels, with 264.49±18.50 µmol l^−1^ for ALAN-exposed nestlings compared with 338.46±16.95 µmol l^−1^ for dark-night nestlings before the immune challenge (i.e. day 13) (*P*=0.017). Furthermore, in contrast to our predictions, nitric oxide levels were lower after the immune challenge for both ALAN-exposed and dark-night birds compared with those before the immune challenge (ALAN: 198.36±11.54 µmol l^−1^, dark-light: 155.61±9.53 µmol l^−1^; *P*<0.001, respectively). Following immune challenge, nitric oxide levels did not differ between dark-night and ALAN-exposed birds (*P*=0.4).

**Table 1.**
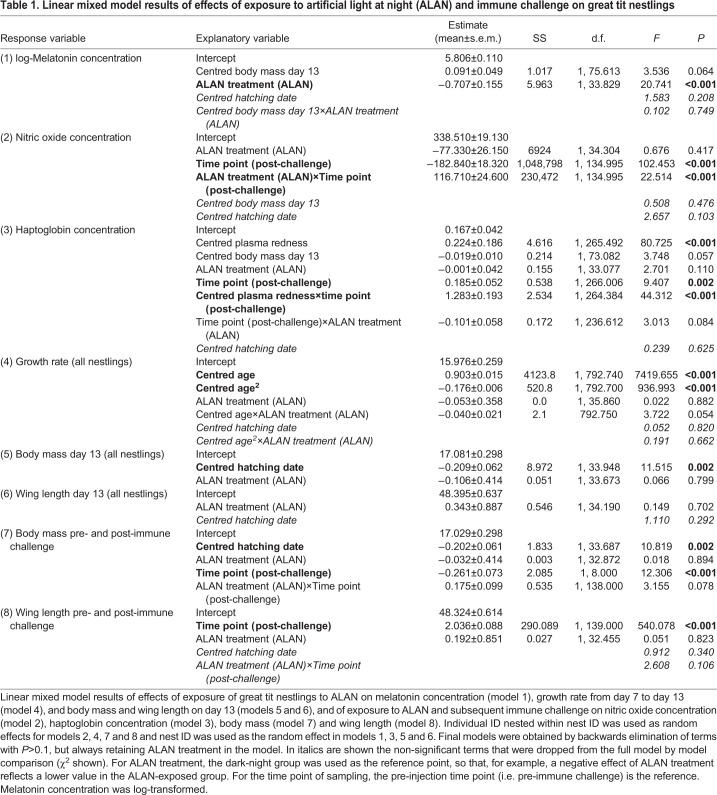
**Linear mixed model results of effects of exposure to artificial light at night** **(ALAN) and immune challenge on great tit nestlings**

**Fig. 3.**
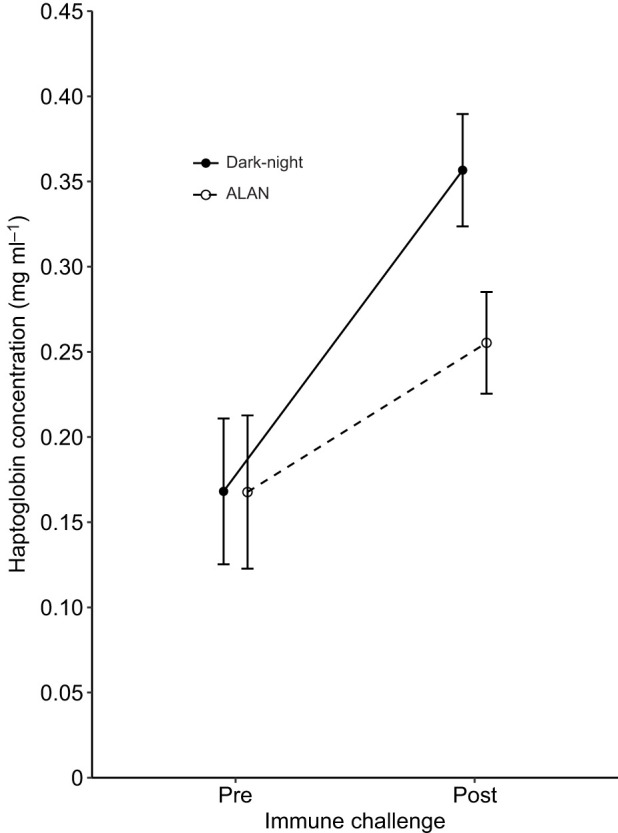
**Effect of exposure to ALAN and immune challenge on haptoglobin concentration in great tit nestlings.** Nestlings were assigned to dark-night (*N*=63) or ALAN (*N*=76) treatment and then subjected to an immune challenge (LPS injection). Estimated marginal means±s.e.m. calculated from the final model (Table 1) are shown.

Nestling haptoglobin levels tended to react differently to the immune challenge depending on whether they were ALAN exposed or not (ALAN *N*=76 nestlings of 19 broods, dark-night *N*=63 nestlings of 18 broods; Table 1, model 3; Fig. 3). The slopes of the response following an immune challenge marginally differed between the dark-night and ALAN treatment groups, with a steeper increase of haptoglobin levels for dark-night nestlings compared with ALAN-exposed nestlings (*P*=0.084; Table 1, model 3; Fig. 3). *Post hoc* pairwise comparisons indicated that dark-night nestlings had higher haptoglobin levels after the immune challenge than before (*P*=0.002), which was not the case for ALAN-exposed nestlings (*P*=0.3). Additionally, haptoglobin levels between dark-night and ALAN-exposed nestlings did not differ before the immune challenge (ALAN and dark-night: 0.13±0.01 mg ml^−1^; *P*=1), but tended to differ after the injection (ALAN: 0.52±0.06 mg ml^−1^, dark-night: 0.63±0.11 mg ml^−1^; *P*=0.067). Plasma redness was dependent on the time point of sampling, with more red plasma (i.e. higher absorption values) after injection than before (Table 1, model 3).

While ALAN exposure and the immune challenge affected haptoglobin and nitric oxide levels differently, concentrations of the two markers before as well as after the immune-challenge were not significantly correlated with each other (pre-injection *r*=−0.05, *P*=0.53; post-injection *r*=0.06, *P*=0.47).

### Biometrics

**Fig. 4.**
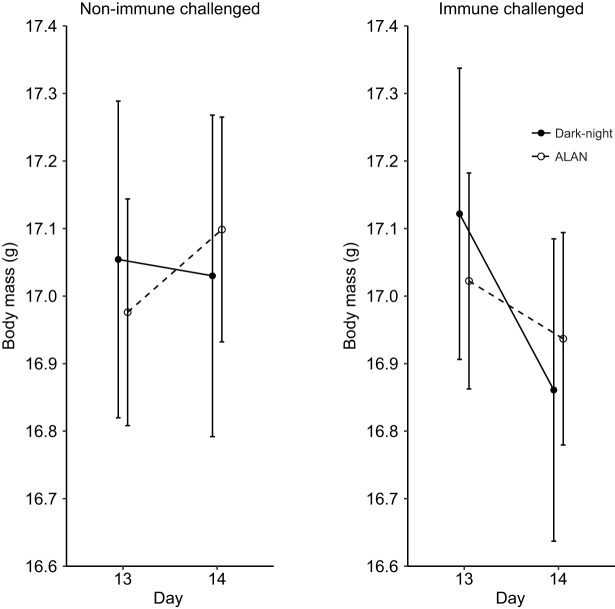
**Effect of exposure to ALAN and immune challenge on body mass in great tit nestlings.** One group of nestlings was subjected to an immune challenge (LPS injection; right), the other was not (left), and body mass was assessed on day 13 and day 14 (i.e. pre- and post-immune challenge for the nestlings on the right). Dark-night: total *N*=143, LPS *N*=64 nestlings; ALAN: total *N*=123, LPS *N*=76 nestlings. Means±s.e.m. of raw data are shown.

The increase in nestling body mass from 7 to 13 days did not differ between ALAN-exposed and dark-night nestlings for the quadratically fitted growth curve (Table 1, model 4), but ALAN-exposed nestlings tended to grow at a slower rate compared with dark-night nestlings when a linear growth curve was fitted (Table 1, model 4) (ALAN *N*=143 nestlings of 19 broods, dark-night *N*=124 nestlings of 18 broods). Neither body mass (ALAN *N*=143 nestlings of 19 broods, dark-night *N*=123 nestlings of 18 broods; Fig. 4) nor wing length (ALAN *N*=140 nestlings 19 broods, dark-night *N*=123 nestlings of 18 broods) at day 13 was affected by ALAN exposure (Table 1, models 5 and 6, respectively). Nestlings that hatched later in the season weighed significantly less on day 13 than earlier-hatched nestlings, but hatching date did not have an effect on growth rate or explain variation in wing length at day 13 (Table 1, models 4, 5 and 6).

Among the nestlings that received an immune challenge (ALAN *N*=76 nestlings of 19 broods; dark-night *N*=64 nestlings of 18 broods), nestlings tended to react differently in terms of the change in body mass during the 15–16 h after the immune challenge, depending on whether they were exposed to ALAN or not (Table 1, model 7; Fig. 4). *Post hoc* pairwise comparisons showed that dark-night nestlings had lower body mass after the immune challenge than before (*P*=0.001), which was not the case for ALAN-treated nestlings (all other comparisons *P*>0.5, Fig. 4). Neither ALAN treatment nor the immune challenge, or their interaction had any significant effect on wing length (Table 1, model 8).

## DISCUSSION

In the present study, we exposed wild great tit nestlings for seven nights to ALAN and subsequently subjected them to an immune challenge to test whether the capacity to mount an immune response to a mimicked bacterial infection was constrained by the ALAN exposure. Overall, ALAN exposure altered the immune response, as demonstrated by a differential change in two key inflammatory markers, haptoglobin and nitric oxide. These results suggest that ALAN, at an intensity mimicking an average urban environment, can compromise the ability of nestlings to mount an immune response. In line with other studies, our results suggest that this is mediated, directly or indirectly, by reduced melatonin levels (e.g. Carrillo-Vico et al., 2006; Maestroni et al., 1986; Tan et al., 2010). We found on average 49% lower levels of melatonin during the night in ALAN-exposed nestlings compared with dark-night birds. This reduction of melatonin concentration indicates that the ALAN exposure treatment of 3 lx was sufficient to function as an endocrine disruptor, leading to downstream effects on physiology.

We found that ALAN-exposed nestlings reacted to an immune challenge with a smaller change of nitric oxide levels between the pre- and post-immune challenge samples than dark-night nestlings. In contrast to the pre-immune challenge (baseline) haptoglobin levels, we found that the initial concentrations of nitric oxide were lower for the ALAN-exposed nestlings than for the dark-night nestlings, indicating that ALAN exposure is able to change baseline levels of certain immune indices (see also Raap et al., 2016a). Nitric oxide has generally been found to increase after an immune challenge (Coleman, 2001; Wink et al., 2011). However, we found an unexpected overall decrease in nitric oxide levels after the immune challenge in both treatment groups. We can only speculate about the reasons for our findings. First, induced nitric oxide production is considered to react quickly to an immune challenge. By sampling 15.5 h after the immune challenge, it is possible that we missed the peak of nitric oxide production. However, this does not explain why post-immune challenge levels were below pre-immune challenge levels. Another explanation could be a mechanism to prevent oxidative damage. Haptoglobin and other antioxidants might have scavenged pro-oxidant molecules that were generated during the acute phase of the inflammation process (Halliwell and Gutteridge, 2015). Moreover, as post-immune challenge haptoglobin levels tended to be higher, and melatonin levels were markedly higher, in dark-night birds compared with ALAN-exposed nestlings, one could expect that more nitric oxide was removed from their body than in ALAN-exposed birds. However, this hypothesis is not well supported, as we found no correlation between haptoglobin and nitric oxide levels within birds, and therefore we cannot assume a direct effect of haptoglobin on nitric oxide levels (see also Raap et al., 2016a). Nevertheless, other antioxidants might have been involved and have been more abundant in the dark-night nestlings, as melatonin is known to positively influence the production of antioxidants (reviewed in Rodriguez et al., 2004). Raap et al. (2016b) did not find any differences in the concentrations of antioxidants such as glutathione, total antioxidant capacity and three antioxidant enzymes in great tit nestlings, though ALAN exposure only lasted two nights. Lastly, *in vitro* studies of murine macrophages have revealed that high doses of melatonin suppress the induced production of nitric oxide, presumably to prevent oxidative damage (Gilad et al., 1998). This could offer one explanation for our observed decrease in nitric oxide levels after an immune challenge and for the stronger response in dark-night compared with ALAN-exposed nestlings. However, further investigation of the dynamics of nitric oxide production and removal is needed.

Although we predicted a weaker increase of haptoglobin levels in ALAN-exposed nestlings in response to the immune challenge, we found limited evidence that haptoglobin levels were modified by ALAN exposure. ALAN-exposed nestlings tended to have a smaller increase of haptoglobin levels compared with dark-night nestlings. Previous laboratory experiments on light exposure and immune responses suggested that animals kept for several weeks under mostly constant light conditions show weaker immune responses, which corroborates our findings in a wild population (crickets: Durrant et al., 2020; chickens: Kirby and Froman, 1991; quails: Moore and Siopes, 2000). While baseline haptoglobin levels were not affected by the ALAN exposure treatment, there was a trend that our ALAN treatment groups differed post-immune challenge. Our result of unchanged baseline levels is in contrast to findings of an increase in baseline haptoglobin concentrations after an ALAN exposure of two nights in great tit nestlings (Raap et al., 2016a). Whether this difference from our findings can be explained by the difference in exposure time remains to be examined. However, similar to our findings, Siberian hamsters that were exposed to dim night light for 4 weeks exhibited a difference in bactericidal capacity of the blood after an immune challenge, but not in baseline immune function (Bedrosian et al., 2011).

We did not find consistent differences in growth rate or body mass between the ALAN-exposed and dark-night groups at the end of the experiment. This is in contrast to previous findings, where great tit nestlings exposed to ALAN for two nights failed to gain body mass, whereas non-exposed nestlings gained body mass (Raap et al., 2016b). There are two possible explanations for our results: (1) a change in behaviour of the nestlings and/or (2) a change in behaviour of the female. First, ALAN exposure could have also affected nestling behaviour, as nestling activity and begging behaviour have been shown to increase in response to ALAN exposure (Raap et al., 2016c). More intense begging behaviour should lead to higher feeding rates by the parents and hence positively affect mass gain of the nestlings (Bengtsson and Rydén, 1983). However, higher activity also increases energy expenditure, which in turn could diminish mass gain and growth (Rodríguez-Gironés et al., 2001; Verhulst and Wiersma, 1997). This could explain why we did not find clear overall differences in nestling mass or growth rate between treatments in our study. Second, nestling body mass is largely governed by parental feeding effort. ALAN exposure could have affected maternal sleep and activity levels (Raap et al., 2016c) and provisioning rates, and thus have had an effect on nestling mass. Indeed, Titulaer et al. (2012) found that females increased the feeding rate during the second half of the nestling phase when exposed to 10 lx of ALAN, though with no effects on nestling mass. We hypothesize that if ALAN had effects on parental feeding behaviour, they were short lived, and the female grew accustomed to the ALAN and resumed normal feeding behaviour.

Female brooding of the nestlings during the night could possibly have led to a shielding from ALAN and decreased the effectiveness of the ALAN treatment. We believe that the nestlings in our experiment were exposed to ALAN for most of the treatment period for the following reasons. First, we found a marked reduction of melatonin levels in ALAN-exposed nestlings, indicating a sufficient exposure to ALAN. Additionally, tit nestlings are effectively homeothermic at 8 days of age, making brooding unnecessary for most of our treatment time (Andreasson et al., 2016). In line with this, female marsh tits, *Poecile palustris*, spending the night in the nest were commonly roosting on the nest rim out of contact with their nestlings (Nilsson and Nord, 2017). Similarly, when performing the immune challenge at day 13, we also occasionally found females present in the nest box, but not sitting on the nestlings. Thus, while the brooding of the female might have ameliorated effects of the ALAN treatment during the early phase of the experiment, we believe that the ALAN exposure was overall effective, especially at the end of the experiment.

Mounting an immune response is associated with costs in terms of resources and energy (Bonneaud et al., 2003), and most animals lose mass after an immune activation as a result of a mixture of increased metabolism and sickness behaviours (Lochmiller and Deerenberg, 2000; Owen-Ashley and Wingfield, 2007).We found a marginally significant difference in mass loss between ALAN-exposed and dark-night nestlings during the immune challenge, with dark-night nestlings losing more mass during the acute-phase response than ALAN-exposed nestlings (Fig. 4). Our findings suggest that the ALAN-exposed nestlings either had less resources available to fight the immune challenge (Norris and Evans, 2000) or alternatively could afford to devote more resources to an immune response and engage in anorexia as part of a stronger acute-phase response (Costantini and Møller, 2009; Hasselquist and Nilsson, 2012; Råberg et al., 1998).

The altered immune response in ALAN-exposed nestlings in combination with the preserved growth rate irrespective of the ALAN exposure treatment may suggest a trade-off between life-history traits. Prioritizing growth and size at fledging is beneficial, as the probability for survival and recruitment increases with fledging mass (Both et al., 1999; Krementz et al., 1989; Monrós et al., 2002). Trade-offs between competing processes, such as growth and immune function, are known to be more evident under harsh or stressful conditions, when resources are limited (Hegemann et al., 2013b; Lindström, 1999; Norris and Evans, 2000; Sheldon and Verhulst, 1996). In urban environments, where not only ALAN is present but also a multitude of stressors such as air and noise pollution and reduced food availability and quality (Shanahan et al., 2014; Halfwerk and Slabbekoorn, 2015; Isaksson and Bonier, 2020; Sprau et al., 2017), it is likely that such trade-offs have larger effects on organismal performance and fitness than in rural environments.

In conclusion, we provide mechanistic evidence for the effect of ALAN on the physiology of a wild animal. Exposure to ALAN disrupts circadian rhythms by drastically reducing melatonin levels, with downstream effects on immune function and potential effects on reproductive timing and migration in adulthood. Taken together, the fact that we did detect different physiological responses between ALAN-exposed and dark-night birds, both at baseline levels and after an immune challenge, warrants further investigation of the impact of ALAN, along with other stressors, in an urban setting.
